# Ethics

**Published:** 1874-11

**Authors:** 


Ethics.—* * * There were no wranglings about "*ethics*," but a seeming realization on the part of every one that ethics was something that every one could make for themselves. And by this we do not mean in a literal sense that every one was to follow loosely his own ethics; but that all had a higher law within their own consciousness, that they had followed and were willing to follow.

Gil Blas said, and which we believe was a Spanish adage, that "you cannot make a silk purse out of a sow's ear." Now let us reverse this, and ask ourselves the question whether we can make a "sow's ear" out of a "silk purse." In other words, the man who has within himself a correct principle, and always follows it, whether he will ever descend to "tricks," even though there be no written law. And again, whether any written law will ever elevate men so that their fellow practitioners shall have no complaint to make of them. And whether any law, outside of the conscience, can be made which "tricksters" cannot easily avoid. Our opinion is, and has been for a long time, that we do not want so much ethical discussion or amendation as we do the elevation, by example and education of a higher order of professional men. This seemed to us to be the type of men which met at Columbia, representing the South Carolina Medical Association.—*Charleston Medical Journal and Review.*

